# Linking oceanic variability, euphausiid hotspot persistence, and marine predator distribution along Canada's west coast

**DOI:** 10.1002/eap.70141

**Published:** 2026-01-12

**Authors:** Rhian Evans, Stéphane Gauthier, Clifford L. K. Robinson, Philina A. English, Chelsea Stanley, Brianna M. Wright, Linda Nichol

**Affiliations:** ^1^ Fisheries and Oceans Canada Institute of Ocean Sciences Sidney British Columbia Canada; ^2^ Swire Institute of Marine Science and Area of Ecology and Biodiversity School of Biological Sciences, The University of Hong Kong Hong Kong China; ^3^ Department of Biology University of Victoria Victoria British Columbia Canada; ^4^ Fisheries and Oceans Canada, Pacific Biological Station Nanaimo British Columbia Canada

**Keywords:** climate change, co‐occurrence, euphausiids, hake, krill, oceanography, spatiotemporal distribution, trophic relationships, whales, zooplankton

## Abstract

Understanding patterns of habitat use across trophic levels and the physical drivers of multispecies aggregations is essential to inform ecosystem‐based management. To achieve this, we quantified the spatial distribution and co‐occurrence of hotspots (defined using the Getis‐Ord statistic) for euphausiids and nine of their commercially important fish and whale predators on the west coast of Canada during summer. We first developed fine‐scale spatiotemporal distribution models of euphausiids and Pacific hake using high‐resolution acoustic data from coast‐wide surveys conducted between 2007 and 2018. We found that the spatiotemporal distribution of hotspots of euphausiids and hake was variable between years with low direct overlap (apart from 2017). The summer of 2015, during the 2014–2016 marine heatwave event, was a particularly anomalous year, as euphausiids and hake showed spatial mismatch in their biomass hotspot distributions. For the other eight predator species, predictions from published species distribution models were used to identify spatial hotspots as an average across years. Co‐occurrence patterns were associated with the depth gradient across the shelf and slope and along the canyon and sea valley systems that characterize the Pacific coast of Canada. One assemblage was associated with the deeper parts (200–1000 m+) of the continental slope (euphausiids, hake, redbanded rockfish, sablefish, Pacific ocean perch, and humpback and fin whales) and a different assemblage (redstripe and yellowtail rockfish, and dogfish) was associated with the shallower shelf regions. Important ecological areas with co‐occurring multispecies hotspots occurred along the west coast of Vancouver Island, the sea valleys of Queen Charlotte Sound, and the northwest coast of Haida Gwaii. Our results identify areas where multiple species aggregate, which can inform better management and hopefully protection of these regions that support complex food webs, commercial species, and large predators, and are therefore essential for overall ecosystem health.

## INTRODUCTION

In the northeast Pacific Ocean euphausiids play a key role in the transfer of energy from primary productivity to higher‐order predators (Abraham & Sydeman, 2004; Robinson, 2000; Siegel, 2000). In spring and summer, euphausiids form large, dense swarms along the continental shelf and along the edge of canyon systems where current shear is reduced and a balance between surface and deep currents means they can maintain their horizontal position more efficiently (Cimino et al., 2020; Santora et al., 2011, 2018). In a patchy marine landscape, areas of retention which concentrate primary and secondary production are often referred to as biomass “hotspots” (Benoit‐Bird et al., 2013; Mackas et al., 1997; Simard & Mackas, 1989). Foraging marine predators are attracted to these areas, leading to large multispecies aggregations and the development of important foraging regions which may persist in space and time (Davoren, 2013; Gende & Sigler, 2006; Mackas et al., 1997; Rockwood et al., 2020; Sigler et al., 2012; Simard & Mackas, 1989). Environmental management now focuses on whole‐system health with a focus on maintaining biodiversity (Curtin & Prellezo, 2010; Palumbi et al., 2009). Areas with multispecies aggregations of secondary producers, fish and whales, would therefore be excellent candidates for protection aimed at achieving sustainable management of marine ecosystems.

In the northeast Pacific, euphausiids appear to be one of the largest factors influencing interannual variability in the abundance of their predators (Ware & Thomson, 2005), a large number of which are commercially important (Robinson, 2000; Robinson & Ware, 1994). In this region, relatively few fish species comprise 80%–90% of all commercial fisheries activity (Beamish et al., 2004), and only two—Pacific halibut (*Hippoglossus stenolepis*) and Pacific cod (*Gadus macrocephalus*)—are not considered to be significantly supported by euphausiids (Best & St‐Pierre, 1986). The other species, Pacific salmon (*Oncorhynchus* spp., five species), Pacific sardine (*Sardinops sagax*), Pacific herring (*Clupea pallasii*), sablefish (*Anoplopoma fimbria*), Pacific ocean perch (*Sebastes alutus*), and Pacific hake (hake–*Merluccius productus*), are all significant euphausiid predators during all or some part of their life cycle (Flinn et al., 2002; Lee & Sampson, 2009; Tanasichuk et al., 1991; Ware & McFarlane, 1995), making euphausiids a key factor in local fisheries sustainability.

The northeast Pacific Ocean along the west coast of Canada is an area of increasing climatic variability, and events such as El Niño and marine heatwaves (MHWs) are becoming more intense and frequent (Oliver et al., 2018; Sydeman et al., 2013). There have also been concurrent changes in local food web structure in recent years (e.g., Jacobsen et al., 2022). Hake is the most economically important fish species on the west coast of North America (Hamel et al., 2015). Hake migrate to the west coast of Canada from California during summer to feed and euphausiids make up the largest proportion of their diet (70%–80% depending on size; Robinson, 2000; Tanasichuk et al., 1991). The extent of the northward migration of adult hake is thought to be positively related to sea temperature at 100 m depth, with a higher proportion of adults migrating farther into Canadian waters during warmer years (Malick et al., 2020). However, the distribution of hake within Canadian waters is extremely variable between years, and it is unknown whether this is also related to variability in euphausiid abundance. Rockfish of the genus *Sebastes* are an important component of the west coast food web, both ecologically and economically (Love et al., 2002). Some species of rockfish, such as redstripe (*Sebastes proriger*) and yellowtail rockfish (*Sebastes flavidus*), and Pacific spiny dogfish (*Squalus suckleyi*) are not currently economically important themselves but are often caught as bycatch as they co‐occur in multispecies aggregations with economically important species (Gertseva & Cope, 2011). Some of the more pelagic rockfish species are also significant euphausiid predators, such as redbanded (*Sebastes babcocki*), redstripe and yellowtail rockfish, and Pacific ocean perch (Karinen & Wing, 1987; Lee & Sampson, 2009; Yang, 2006). However, how the distribution of euphausiids impacts that of rockfish is understudied in this region. Rockfish abundance and size‐at‐age are declining along the Pacific coast, due to fishing pressures and bycatch impacts (Eckert et al., 2018; Favaro et al., 2010). Similarly, a relatively high number of other economically important stocks, such as Pacific spiny dogfish, Pacific herring, and Pacific sardine have been declared overfished in the past and some are still considered overexploited (Cleary et al., 2010; Punt, 2011). In addition to these direct anthropogenic pressures, all of these species are also vulnerable to changes in temperature, currents, and oxygen levels along the BC coast related to climate change (Talloni‐Álvarez et al., 2019; Zwolinski & Demer, 2012). Conversely, post‐whaling era humpback whale (*Megaptera novaeangliae*) and fin whale (*Balaenoptera physalus*) populations have been recovering in recent years and represent a potentially increasing top‐down pressure on euphausiids. However, it is unknown how the summertime distribution of whales across the shelf relates to that of euphausiids as a key prey item.

Much of our knowledge of how the abundance and distribution of euphausiids effects the distribution of commercially and ecologically important species on this coast is 20–30 years old (Mackas & Galbraith, 1992; Mackas et al., 1997; McFarlane et al., 1997; Robinson, 2000; Robinson & Ware, 1994; Tanasichuk, 2002—but see Godefroid et al., 2019; Phillips et al., 2023). Marine spatial planning, such as marine‐protected area (MPA) designation, relies on detailed knowledge of the distribution and connectivity of key biological hotspots, and how these change over time (Santora & Sydeman, 2015). Our overarching aim was to quantify the co‐occurrence of euphausiids and a suite of their predators, to identify important regions supporting fisheries and biodiversity. To achieve this, we first required a more detailed understanding of the relationship between hotspots of euphausiids and a main predator, Pacific hake (hereafter “hake”). We used spatiotemporal geostatistical models to predict the distribution of euphausiids and hake (Shelton et al., 2014; Thorson et al., 2015; Ward et al., 2015) using high spatial resolution acoustic data from surveys along the Canadian west coast since 2007.

Our second aim was to assess co‐occurrence of important species across the whole system and spanning trophic levels, using the results of published species distribution models for species identified by Beamish et al. (2004), that had sufficient data available and feed on euphausiids: Pacific ocean perch, Pacific spiny dogfish and sablefish. Other euphausiid predators were also considered if they were important in the ecosystem; we included three other rockfish species mentioned previously (restripe, redbanded, and yellowtail) and two whale species (humpback and fin). Fish distribution models were based on empirical data collected during regional surveys of the west coast of Canada over the same timespan as the data for euphausiids and hake. Together with biomass and distribution predictions for hake and euphausiids developed here, we combined data from all species to quantify overlap in the distribution of hotspots of biomass for each species and identify areas that supported multispecies aggregations. Pacific herring, sardine, and salmon are also important euphausiid predators but were not included in this study due to data availability or model constraints.

## METHODS

### Data sources and analyses

**FIGURE 1:**
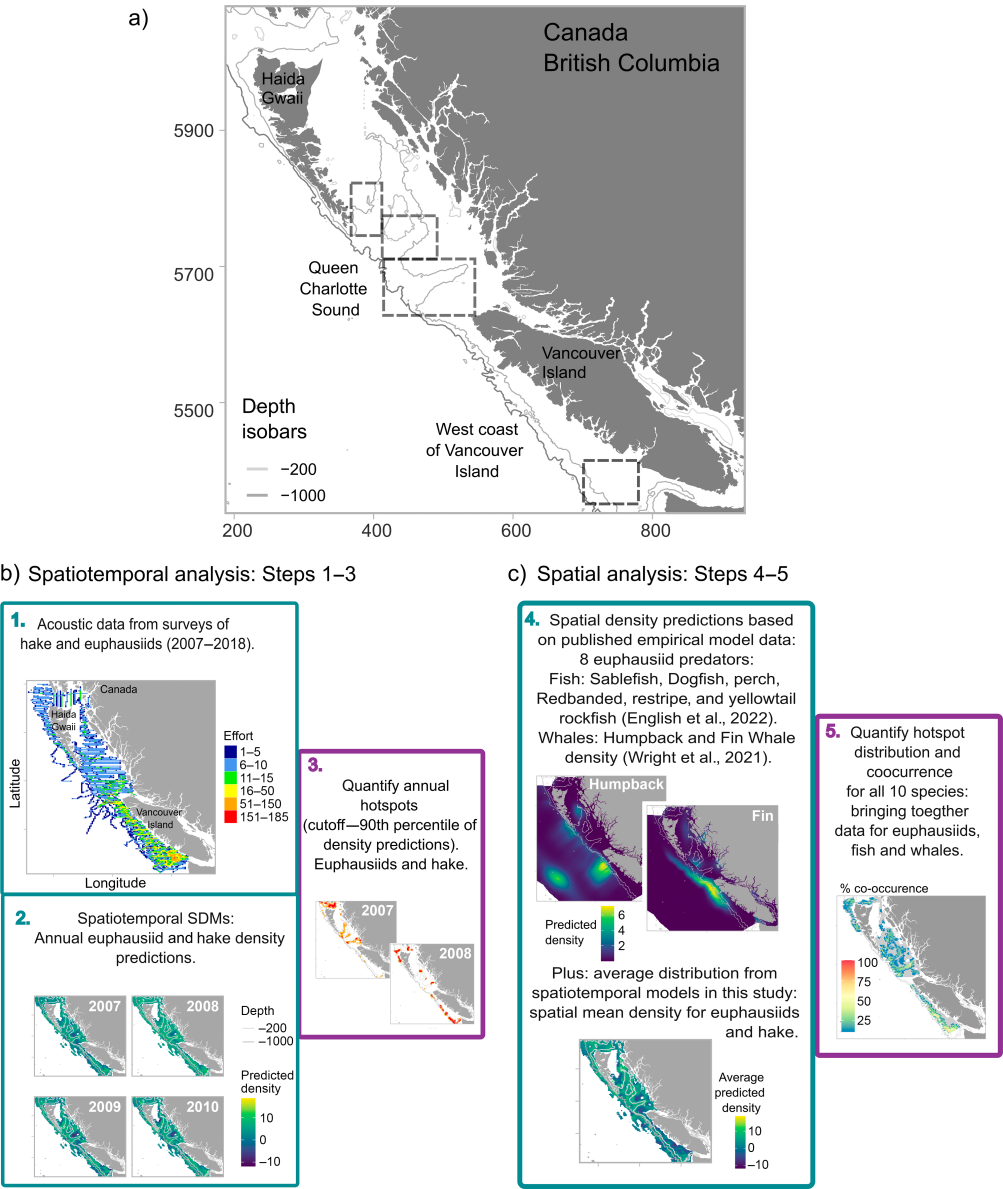
(a) Map of the study region showing location of important regions mentioned in the text. Panles (b) and (c) show our workflow outlining the main steps involved in the study. (b) Steps 1–3 detail empirical data distribution for hake and euphausiids, building the spatiotemporal species distribution models (SDM), and quantifying the overlap of biomass hotspots of the two species across years. (c) Steps 4–5 detail utilizing predictions from published empirical models for six other species of fish, and two whale species, plus mean predictions from SDMs analyzed here for hake and euphausiids, to quantify the co‐occurrence of biomass hotspots across 8 species (excluding whales) for the continental shelf along the west coast of Canada.

We used a multilayered approach for integrating multiple species, data sources, and model outputs. The analytical steps are illustrated in Figure 1 as a guide, along with a map of the study region (the west coast of Canada). We first developed fine‐scale spatiotemporal models for hake and euphausiids and analyzed overlap between hotspots of biomass of these two species (see *Spatiotemporal datasets from raw data: Euphausiid and hake densities* and *Spatiotemporal euphausiid and hake distribution modeling*). Hake is considered the most significant predator of euphausiids along this coast. Then, we extracted biomass and distribution information from published spatial models for the other euphausiid predator species outlined above to create spatial maps of occurrences for euphausiids and a suite of their predators (see *Published spatial models*). Model resolution was matched throughout, and Getis‐Ord statistics were used to quantify the persistence (spatiotemporal datasets), distribution, and mean overlap (spatial datasets) of hotspots.

### Spatiotemporal datasets from raw data: Euphausiid and hake densities

Acoustically derived euphausiid and hake data were collected during multiple acoustic surveys on the west coast of Canada between 2007 and 2018 (see Appendix S1: Figures S1 and S2 for survey years and extents). These surveys included time series from the joint Hake Ecosystem and Acoustic‐Trawl surveys (PHEAT, Gauthier et al., 2020), the La Perouse plankton monitoring surveys (Mackas & Beaugrand, 2010), as well as a range of other programs surveying the outer west coast of Canada in August. Although these surveys covered a range of months, August/September surveys exhibited the most consistent spatial coverage between years and so surveys during this period were taken forward for further analysis. This period also coincides with the timing of peak availability of euphausiids to predators in summer, following spring reproduction (Evans, English, et al., 2021; Evans, Gauthier, & Robinson, 2021), and hake stocks have been feeding on the shelf since spring. The majority of euphausiid biomass in this region constitutes two species, *Euphausia pacifica* and *Thysanoessa spinifera*. The biomass of these two species has been modeled separately for part of this region in two previous studies (Evans et al., 2023; Evans, English, et al., 2021; Evans, Gauthier, & Robinson, 2021). As we are interested in the euphausiid population as an energy‐rich prey base supporting predator distributions in this study, both species were treated together from the acoustically derived index, as both are important in the diets of local predators.

For both euphausiids and hake, all survey vessels collected data from calibrated Simrad EK60 scientific echosounders operating with, at a minimum, 38 and 120 kHz split‐beam transducers (ES38‐B and ES120‐7 series); however, many also included 18, 70, and/or 200 kHz. Both hake and euphausiid aggregations were detected acoustically and hake were verified to species level using midwater trawls. For euphausiids, only data collected during daylight hours from 50 m below the surface to 300 m depth were used to estimate aggregations, while for hake, data from the whole water column were used (based on echosign detection and trawl‐verified aggregations). This matched the known vertical distribution of both groups during daylight.

Acoustic raw data were processed using Echoview (Echoview Pty Ltd., Hobart, Australia) and EchoviewR (Harrison et al., 2015), an R package to interact with Echoview. Acoustic backscatter from adult euphausiids were detected based on the difference in mean volume backscattering strength (MVBS or *S*_*v*_ decibels relative to 1 meter) between two frequencies (38 and 120 kHz) measured in 40 pings by 10‐m cells (MVBS_120–38_). MVBS_120–38_ values were filtered to select cells within the range of 10.0–16.3 dB. These values were derived from acoustic measurements made on net‐verified aggregations of adult *E. pacifica* and *T. spinifera* on the west coast of Canada (see Phillips et al., 2022 for more details). The nautical area scattering coefficient (NASC, in square meters per square nautical mile), which is a proxy for biomass density, was extracted from echograms on all surveys falling within the months of interest in each year sampling took place (see Appendix S1: Figure S1 and was averaged along each 0.5‐nmi section of transect. Euphausiid data were available across more surveys than hake data, which were available from the joint PHEAT surveys only following coast‐wide coverage of trawl‐verified aggregations of adults aged two and up. Acoustic NASC will be referred to as “density” from this point forward.

### Spatiotemporal euphausiid and hake distribution modeling

#### Spatiotemporal environmental predictors

We used 10 environmental predictors extracted from the Regional Ocean Modeling System (ROMS) developed for the British Columbia shelf and slope (Peña et al., 2019), to estimate the biomass and distribution of euphausiids and hake. The model is a circulation model which is an extension of the Masson and Fine (2012) application of the ROMS (Haidvogel et al., 2008), coupled with a Nutrient‐Phytoplankton‐Zooplankton‐Detritus (NPZD) ecosystem model (Peña et al., 2019). The model domain includes the entire Canadian Pacific coast (~55° N–~46° N) and has a spatial resolution of 0.04°. The model has undergone extensive validation for all of the variables included in this analysis (Masson & Fine, 2012). More details about the model can be found in Peña et al. (2019).

Values were extracted as monthly means for each variable between 2007 and 2018 in August when most of the euphausiid and hake sampling occurred. Predictor variables were chosen based on importance, as evaluated by a review of euphausiid species distribution modeling on the west coast of North America (Evans, English, et al., 2021; Evans, Gauthier, & Robinson, 2021). This review also indicated that euphausiids exhibit a lagged response to some environmental conditions. For example, spring primary production cycles mediate the amount of secondary production and would therefore be a factor influencing euphausiid, and perhaps euphausiid predator, density, and distribution in summer. Therefore, we tested models estimating the distribution of euphausiids and hake including environmental predictor variables from both the previous spring (May) and conditions at the time of sampling (August) for each year.

Predictor variables tested included: water temperature (in degrees Celcius) at the surface and at 150 m, salinity, dissolved oxygen concentration (in millimoles per cubic meter), density (in kilograms per cubic meter), and the depth‐integrated production of large phytoplankton (milimoles of Nitrogen per square meter per day). East–west (u) and north–south (v) currents (in meters per second) were also extracted and included to represent upwelling. A proxy of stratification was calculated as the difference in density between 50 m depth and the surface (in kilograms per cubic meter). Water depth (in meters) was the only static predictor in all models. A mask was applied to the model domain to exclude data from inlets, as data from the ROMS model in these areas exhibited inflated error and were not of interest (Peña et al., 2019). We projected all covariates onto a grid of equal sized cells (Universal Transverse Mercator [UTM] zone 9), with the same dimensions as the original ROMS grid (3 × 3 km resolution). For each euphausiid or hake density value along transects, we extracted covariates from the corresponding square in the prediction grid for May and August in each year that surveying took place. Both datasets had many observations—the euphausiid model had *n* = 54,491 observations, and the hake model had *n* = 23,847 observations.

#### Spatiotemporal statistical framework—Euphausiids and hake

Acoustically derived density data have very high spatial resolution. Because of this, and because each NASC value represents the value of a small section of a continuous transect, the data exhibited very high spatial autocorrelation. Due to survey design and conditions, the locations of some observations differed between years. We used a specialized modeling framework to account for these challenges, encompassing a geostatistical model with Gaussian Markov random fields to approximate unexplained spatial variation between sampling locations over time (Lindgren et al., 2011; Thorson et al., 2015; Ward et al., 2015). Latent spatial and spatiotemporal environmental variability were accounted for through a spatial and spatiotemporal random effects structure. Our implementation included a mesh with 800 triangulated cells with vertices known as knots, the location of which was calculated using a k‐means cluster algorithm (Anderson et al., 2019; Anderson & Ward, 2019; Shelton et al., 2014). Bilinear interpolation was then used to estimate the spatial random effects between knot locations at both actual observations (>25,000 values representing 0.5‐nmi sections of transect) and other locations. Through these random fields, the model estimated both spatial patterns, which are consistent through time (spatial variability, e.g., variability associated with depth), and spatial patterns which are not consistent through time (spatiotemporal variability, e.g., variability associated with dynamic oceanic processes). The spatiotemporal fields followed a stationary autoregressive process with a first‐order correlation structure (AR1), giving equal spacing between events (years, in the case of our model). The model therefore assumes that closely distributed (in both space and time) density values are more similar than values farther away from one another.

Models were fit using a generalized additive mixed model (GAMM) framework implemented through the R package “sdmTMB” (Anderson et al., 2022). This package incorporates both integrated nested Laplace approximation (INLA; Rue et al., 2009) and Template Model Builder (TMB; Kristensen et al., 2016) to fit the models by maximum marginal likelihood. In this case, species density was modeled as both a function of the environmental variables, and the unmodelled or “latent” spatial and temporal variability mentioned above. All environmental effects were fit with penalized splines constructed in sdmTMB using the function “smooth2random()” from the “mgcv” package (Wood, 2017). All spatiotemporal GAMMs used a Tweedie distribution observation likelihood (Tweedie, 1984) with a log link, due to the continuous and zero‐inflated nature of the datasets. An additional random intercept of year was included to give the model flexibility to allow some variation in overall mean density outside of the variability explained by the fixed effects and spatiotemporal random fields.

For both euphausiids and hake, we ran three sets of models, one modeling summer density as a function of environmental conditions in the previous spring (May predictors), one using predictors from the time of sampling in August, and one using a mix of May and August predictors. Due to concurvity issues between some predictors (Ramsay et al., 2003); for example, sea surface temperature (SST) and surface density, only uncorrelated variables were tested in the same model (assessed as having an *r* ^2^ lower than 0.7). The most parsimonious combination of variables was chosen based on the lowest Akaike information criterion (AIC) and residual plots. AIC indicates the best model in terms of explaining the most amount of variability in the underlying data using the fewest possible independent variables (Burnham & Anderson, 2004). The August models performed consistently better than the May models for both euphausiids and hake (data not shown), so we focused on the addition of one lagged covariate at a time into the models with all other covariates measured at the time of sampling (e.g., May SST with the other variables from August) to see if the timing of individual variables differed in their influence on euphausiid and hake density, assessed through an improved model fit. We ensured model convergence by checking that the log‐likelihood gradient with respect to all fixed effects was <0.01, that the Hessian matrix was positive definite, and that no random field marginal SDs were estimated as <0.01. See Appendix S1: Tables S2–S5 for full covariate selection. Table 1 gives the final model structures for the summer model and the mixed‐season model. Densities of both hake and euphausiids were predicted over the same grid as used for ROMS covariates (3 × 3 km grid) for each year that sampling occurred, to give spatiotemporal estimates of density for both euphausiids (2007–2018) and hake (biannually between 2007 and 2018 plus 2012). We plotted observed versus predicted densities (both in log space as log(*x* + 0.01)) and calculated the *r* ^2^ of this relationship for both models to assess their overall predictive power (Appendix S1: Figure S3). Once the top model was selected, we reduced the number of knots to a cutoff of 15 km to investigate how much the marginal *r* ^2^ (representing variance explained by the covariates) changed with a more simplified spatial field.

**TABLE 1:** Best model with August predictors and the final model with predictors from both May and August (mixed‐season model) chosen by Akaike information criterion (AIC) for prediction of summer (August) acoustic density of euphausiids and Hake.

Model	Covariates	Model structure (after covariate selection)	AIC	*r* ^2^ (800 knots)	*r* ^2^ (15 km cutoff)
Euphausiid	August	~ factor(year) + s(log depth, *k* = 3) + s(SST, *k* = 3) + s(stratification, *k* = 3) + s(Oxygen 150 m scaled, *k* = 3) + s(U current, *k* = 3) + s(current speed, *k* = 3)	Δ34.4		
	Mixed‐season model	~ factor(year) + s(log depth y, *k* = 3) + s(may SST, *k* = 3) + s(aug stratification, *k* = 3) + s(may Oxygen 150 m, *k* = 3) + s(aug U current, *k* = 3) + s(may Current speed, *k* = 3)	Δ0	0.91 (0.81)	0.88 (0.78)
Hake	August	~ factor(year) + s(log depth, *k* = 3) + s(temperature 150 m, *k* = 3) + s(stratification, *k* = 3) + s(Oxygen 150 m, *k* = 3) + s(production large phytoplankton, *k* = 3) + s(U current, *k* = 3) + s(Current speed, *k* = 3)	Δ36		
	Mixed‐season model	~ factor(year) + s(log depth, *k* = 3) + s(may temperature 150 m, *k* = 3) + s(aug stratification, *k* = 3) + s(may Oxygen 150, *k* = 3) + s(may production large phytoplankton, *k* = 3) + s(aug U current, *k* = 3) + s(aug Current speed, *k* = 3)	Δ0	0.95 (0.94)	0.94 (0.87)

*Note*: The *r* ^2^ was calculated for top covariate models with both 800 knots and a more simple mesh with a 15‐km cutoff. The conditional *r* ^2^ for both types of models is shown, which is the overall variance explained by the model, with the spatiotemporal *r* ^2^ shown in brackets (the variance explained by the spatial and spatiotemporal fields).

Abbreviation: SST, sea surface temperature.

### Published spatial models

We utilized spatial distribution data predicted from several published spatial models in the same region to expand the number of euphausiid predators that could be included in this analysis. We outline these data below. In all cases, when all models are compared, we averaged biomass predictions of all species onto the largest sampling grid (the whale data, which was predicted onto a 5 × 5 km grid). Data time‐range were truncated prior to averaging to that of the dataset with the fewest years of sampling available. For example, when comparing the distribution of euphausiids, hake, and groundfish, groundfish were surveyed in the fewest years; therefore, we only use data from the other two datasets in years where groundfish data were also available. However, when comparing the distribution of euphausiids, fish, and whales, only data from 2018 were considered for all species, matching with the only large‐scale survey for whales.

#### Groundfish

The Fisheries and Oceans Canada synoptic groundfish survey has occurred biannually on the west coast since 2003 with different parts of the coast surveyed between May and August (Anderson et al., 2019). The spatiotemporal biomass densities of numerous demersal fishes were modeled for mature and immature portions of their populations as part of a separate study of the impacts of climate velocities on Pacific groundfish (English et al., 2022). These models use the same underlying spatiotemporal statistical framework as described above, also implemented in sdmTMB, but with the only covariate being a time‐varying quadratic effect of bottom depth (see sect. 2.2 in English et al., 2022). Biomass distribution predictions for adult dogfish; sablefish; ocean perch; and redstripe, yellowtail, and redbanded rockfish were generated from these models using the 2 × 2 km grid (English et al., 2022). For use in this study, we included only density predictions for years and regions where fishery‐independent trawl surveys were conducted and that also matched the temporal resolution (2007–2018) of the euphausiid data (Appendix S1: Figure S4).

#### Humpback and fin whales

Humpback and fin whale distributions were modeled using ship‐based survey data from the Pacific Region International Survey of Marine Megafauna (PRISMM); a large‐scale survey aimed at understanding whale abundance and distribution in Canadian waters (Wright et al., 2021). The survey was conducted between July and September in 2018 only, according to distance sampling protocols (Buckland et al., 2001). Whale sightings were converted to effort‐corrected density predictions for coastal and offshore waters using generalized additive models (GAMs) in relation to static habitat covariates (distance to the 1000‐m contour and sighting locations summarized as easting and northing). Effort‐corrected model predictions of both humpback and fin whale densities were made across a 5 × 5 km grid and were spatial only (see Appendix S1: Figure S5 for predictions). See Wright et al. (2021) for a more detailed overview of the survey design, data processing, and modeling framework.

### Quantifying spatial and spatiotemporal biomass hotspots of euphausiids, fish and whales

The Getis‐Ord statistic (G*i*; Getis & Ord, 1992) was used to quantify the spatial structure and patchiness of both spatiotemporal density predictions (for annual predictions of euphausiids and hake), and spatial density predictions (mean spatial predictions of humpback and fin whale, dogfish, sablefish; ocean perch; and redbanded, redstripe, and yellowtail rockfish). The G*i* statistic (a *Z*‐score) quantifies local clustering relative to the overall spatial mean and SD by calculating the distance among cells over which relative biomass values are correlated (Dorman et al., 2015; Kuletz et al., 2015). To inform the G*i* statistic, the spatial neighborhood needs to be determined for each species. Global Moran's *I* process is used to quantify spatial autocorrelation; therefore we used this statistic to first quantify at what distance similarities between biomass values started to decay and to set the nearest neighbor value for the G*i* statistic to be calculated (see Kuletz et al., 2015 for a practical application of this method). For all species, grid squares with G*i Z*‐scores in the 90th percentile were considered hotspots. For euphausiids and hake with spatiotemporal density predictions, *Z*‐scores were calculated across sampling years, meaning that hotspot intensity and distribution were quantified in the context of the whole timeseries, and hotspot intensity could be compared between years. We mapped spatiotemporal hotspots for euphausiids and hake and calculated the percentage (in percentage) overlap between these hotspots for every year that both groups were sampled.

To achieve our second aim, we brought together density predictions from models constructed here (hake and euphausiids) and published density predictions for the other euphausiid predators based on empirical models (described above); six groundfish and two whale species. We calculated spatial biomass predictions of euphausiids and hake by regridding all datasets to the lowest resolution dataset (whale predictions at 5 × 5 km) and taking a mean across years for each grid cell and truncating extents to match the spatial extent of groundfish predictions (which has the smallest area surveyed). The G*i* statistic was then applied to euphausiid and fish datasets using the same method as above to quantify the mean distribution of biomass in the 90th percentile (hotspots) for each species. To examine the distribution of areas of high co‐occurrence of hotspots, we calculated a percentage overlap between fish species' hotspots and euphausiid hotspots.

To examine the links between the spatial distribution of hotspots of fishes and euphausiids with environmental conditions we used redundancy analysis (RDA). RDA is a constrained ordination analysis that is an extension of multiple regression analysis for multivariate data that allows multiple response variables to be “constrained” in a reduced space constructed from the explanatory variables (Rao, 1964). Therefore, it is used to quantify explained variance of the dependent variables by a linear combination of the explanatory variables. In this case, the calculated G*i Z*‐score for each species was the dependent variable representing the distribution of areas of aggregated densities and how they co‐occurred, and the environmental variables were the explanatory or “constraining” variables taken from the ROMS model and used previously in species distribution modeling (SDM, see above). The two whale species were excluded from this RDA as the temporal resolution of the underlying data was different (2007–2018 for euphausiids and fish, 2018 only for whales). However, we quantified the distribution of whale hotspots in the 90th percentile in 2018 with the distribution of hotspots of euphausiids in 2018 only, to highlight regions of importance for whales in the area.

## RESULTS

### Spatiotemporal model fit for euphausiids and hake

The final top models for euphausiid and hake exhibited high conditional *r* ^2^ values of 0.87 and 0.94, respectively. The majority of variance in the underlying data was explained by the spatial and spatiotemporal fields rather than the marginal (covariate) effects (euphausiid marginal effects *r* ^2^ 0.08; hake marginal effects *r* ^2^ 0.05). However, as most of the covariates used in the models were spatiotemporally variable, there was collinearity between the covariates and the spatiotemporal effects used in the model. Therefore, the very low variance explained by fixed effects (covariates) was most likely a product of the model struggling to assign the variation to the mesh or the covariates, demonstrated by the increase in marginal *r* ^2^ as the spatial mesh became simpler (see Table 1). We were mostly interested in models that had high predictive capacity to explore the spatiotemporal distribution of hotspots; therefore, covariate importance was not the focus here.

**FIGURE 2:**
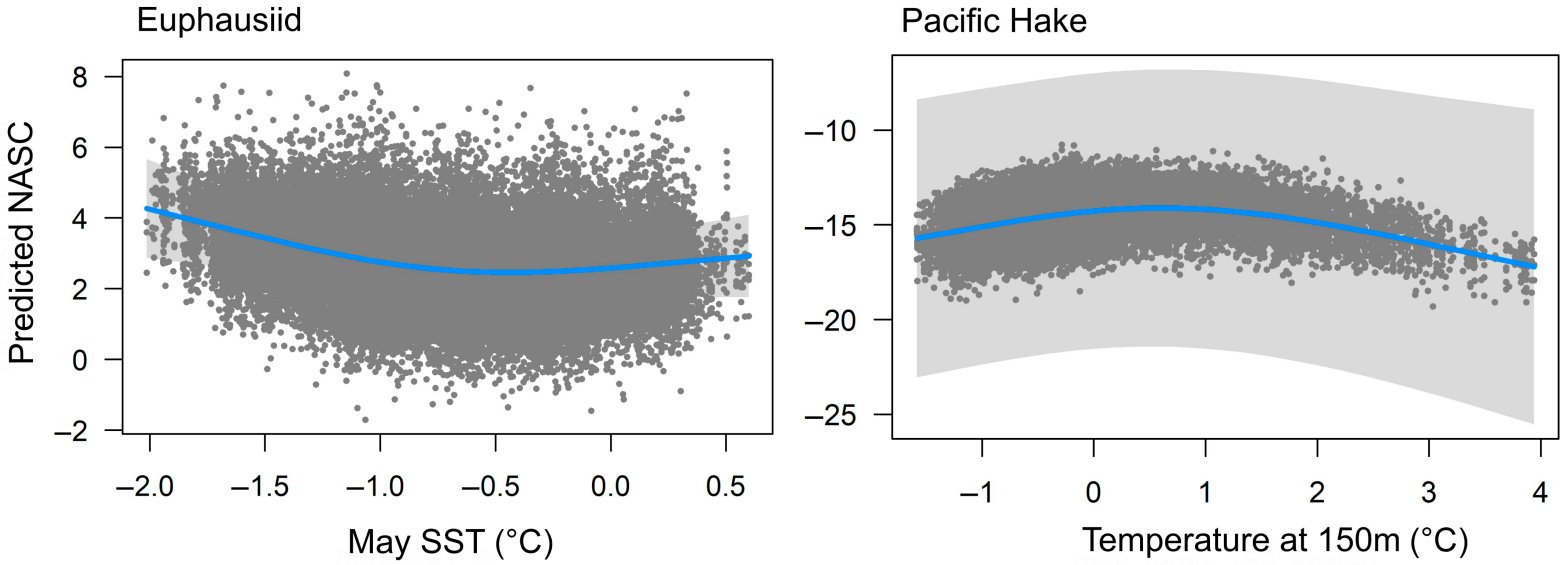
Conditional effects showing the relationship between the temperature variable and the density (represented by nautical area scattering coefficient [NASC] on the log link scale) of euphausiids and hake, showing the relationship with all other variables held at their median. The *X*‐axis represented the range of each predictor in the model scaled by the mean. Shaded sections represent ±95% CIs of the smooth. Background points are the partial residuals of fits.

Euphausiid density was best predicted by a model including bottom depth, SST, water column stratification, oxygen (150 m), U current (E–W), and current speed (see Appendix S1: Tables S2 and S3 for covariate selection). When variables from both time periods were included, spring (May) SST, oxygen, and current speed produced a better model fit compared with models with these variables at the time of sampling in August, whereas summer U current, an indication of upwelling strength, was better correlated with euphausiid density at the time of sampling during summer. Conditional effects indicated a negative relationship between May SST and summer euphausiid biomass: that is, higher temperatures in spring resulted in lower biomass in summer (Figure 2). Including variables from the previous spring into predictions resulted in a delta AIC of >34.

Hake biomass was best predicted by a model including bottom depth, temperature at 150 m, water column stratification, oxygen at 150 m, primary production, U current (E–W), and current speed. The models with mixed August and May covariates outperformed models with only August environmental conditions, resulting in a delta AIC of >36 (Appendix S1: Table S4). May temperature at 150 m, May oxygen (150 m), and May primary production were the best indicators of summer hake density, combined with the water column stratification and current variables at the time of sampling. Conditional effects plots indicated a dome‐shaped relationship between the density of hake in Canadian waters and the temperature at 150 m in summer (Figure 2).

### Spatiotemporal co‐occurrence of hotspots of euphausiids and hake

**FIGURE 3:**
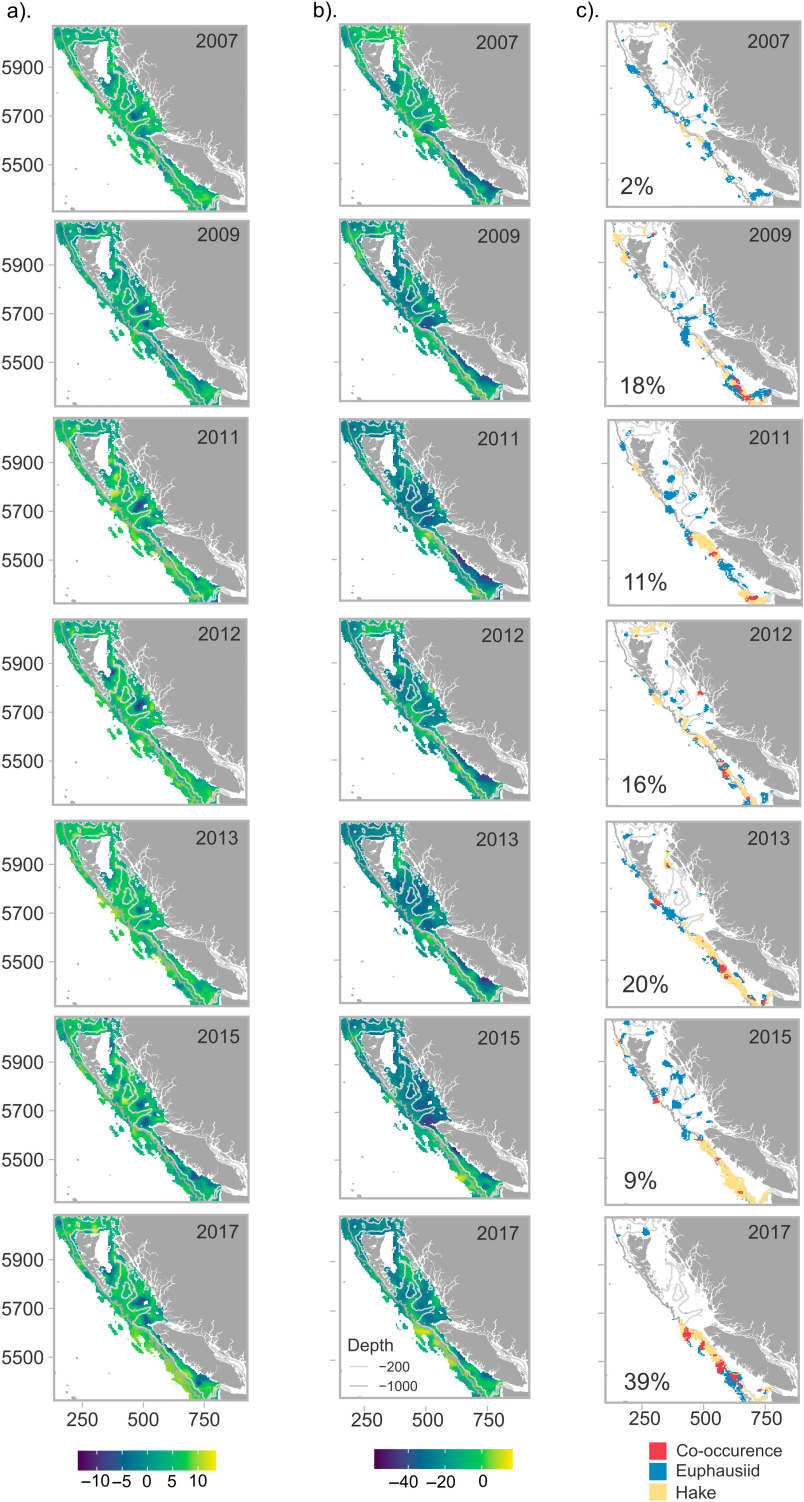
(a) Spatiotemporal predictions of euphausiid nautical area scattering coefficient (NASC, in square meters per square nautical mile), including both fixed effects and random effects. Euphausiid predicted density is shown on the link scale of the Tweedie model (log link). (b) Spatiotemporal predictions of Hake NASC (in square meters per square nautical mile), including both fixed effects and random effects. Hake predicted density is shown on the link scale of the Tweedie model (log link). (c) The estimated spatiotemporal distribution of *Z*‐scores in the 90th percentile for euphausiids (blue) and Hake (yellow) acoustic density, which were considered hotspots for this analysis across 3 × 3 km grid squares. Red areas indicate cells which were hotspots for both hake and euphausiids in that year and so were areas of co‐occurrence. The percentage overlap (proportion of hotspot that is co‐occurrence vs. hotspot that is just one species) is shown for each year. Predictions are shown for years in which both hake and euphausiid were sampled (biannually between 2007 and 2018, plus 2012).

Full spatiotemporal density predictions for hake and euphausiid models are shown in Figure 3a,b. Positive *Z*‐scores in the 90th percentile or greater were considered hotspots. There was high interannual variability in the spatial distribution of predicted hotspots for both euphausiids and hake (Figure 3c). Co‐occurrence between hotspots of hake and euphausiids was lowest in 2007 and 2015 (2% and 9% of total hotspot area, respectively), and highest in 2013 and 2017 (20% and 39% of total hotspot area, respectively). Spatially, predicted hotspots of both euphausiids and hake occurred along bathymetric transition zones, including along the continental shelf boundary between the 200‐ and 1000‐m contours, and along sea canyons and sea valleys (Figure 3c). Areas of hotspot co‐occurrence for hake and euphausiids were usually situated just deeper than the 200‐m contour, with the most consistent area of overlap being the west coast of Vancouver Island (Figure 3c). Co‐occurrence also occurred along the sea valleys of Queen Charlotte Sound, and along the north and west coast of Haida Gwaii (see Figures 1a and 3c).

**FIGURE 4:**
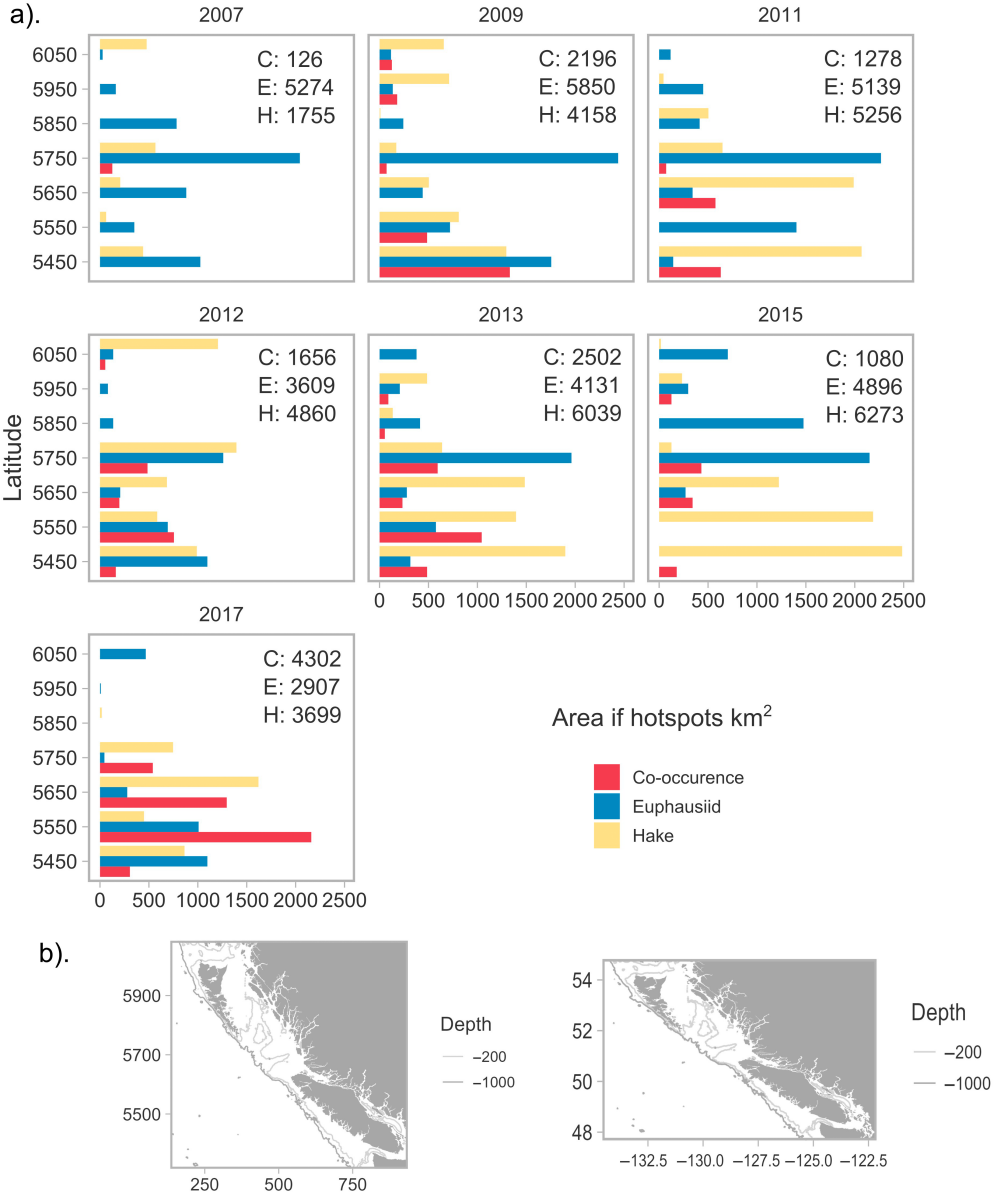
(a) Bar length indicates the predicted size, in terms of area of hotspots (in square kilometers) for euphausiids (blue), hake (yellow), and co‐occurrence of hotspots (red), for each year, binned across 100 km of latitude (*X* and *Y* axis units are given in Universal Transverse Mercator [UTM] zone 9). *Y* axis values indicate the midpoint of binned latitudes. The overall total size of hotspots (in square kilometers) in each year for euphausiids (E), hake (H), and co‐occurrence (C) is also given. (b) Coordinates in UTM (in kilometers, left) and latitude/longitude (in degrees, right) for the study region to aid in interpretation.

Predicted euphausiid hotspots covered the largest area in 2009 (8046‐km combined euphausiid and co‐occurrence Figure 4), while hake hotspots covered the largest area in 2013 (8541 km combined hake and co‐occurrence). The distribution of hotspots by latitude for both hake and euphausiids varied interannually. Aggregations of hake were either evenly distributed across latitudes (2007, 2009, 2012) or centered in the south of the study region off Vancouver Island (2011, 2013, 2015, and 2017; see Figure 4). The size of euphausiid hotspots peaked in most years between 5600 and 5800 km (UTM zone 9, see Figure 4b for latitude and longitude) along depth gradients within Queen Charlotte Sound and along the west coast of Vancouver Island between 5400 and 5600 km. The exception to this was 2015 (Figure 4). Co‐occurrence of both hake and euphausiid hotspots occurred throughout the study region in all years except 2007 (the lowest year of co‐occurrence), 2011, and 2015. 2015 was notable for exhibiting opposing trends in the distribution of hake and euphausiids, with very large euphausiid hotspots centered in the north of the study region but not off the west coast of Vancouver Island, while hake hotspots were not distributed in the north but were centered in the lower study region where euphausiids, their main prey, were absent.

### Spatial distribution and co‐occurrence of hotspots of euphausiids and fish

**FIGURE 5:**
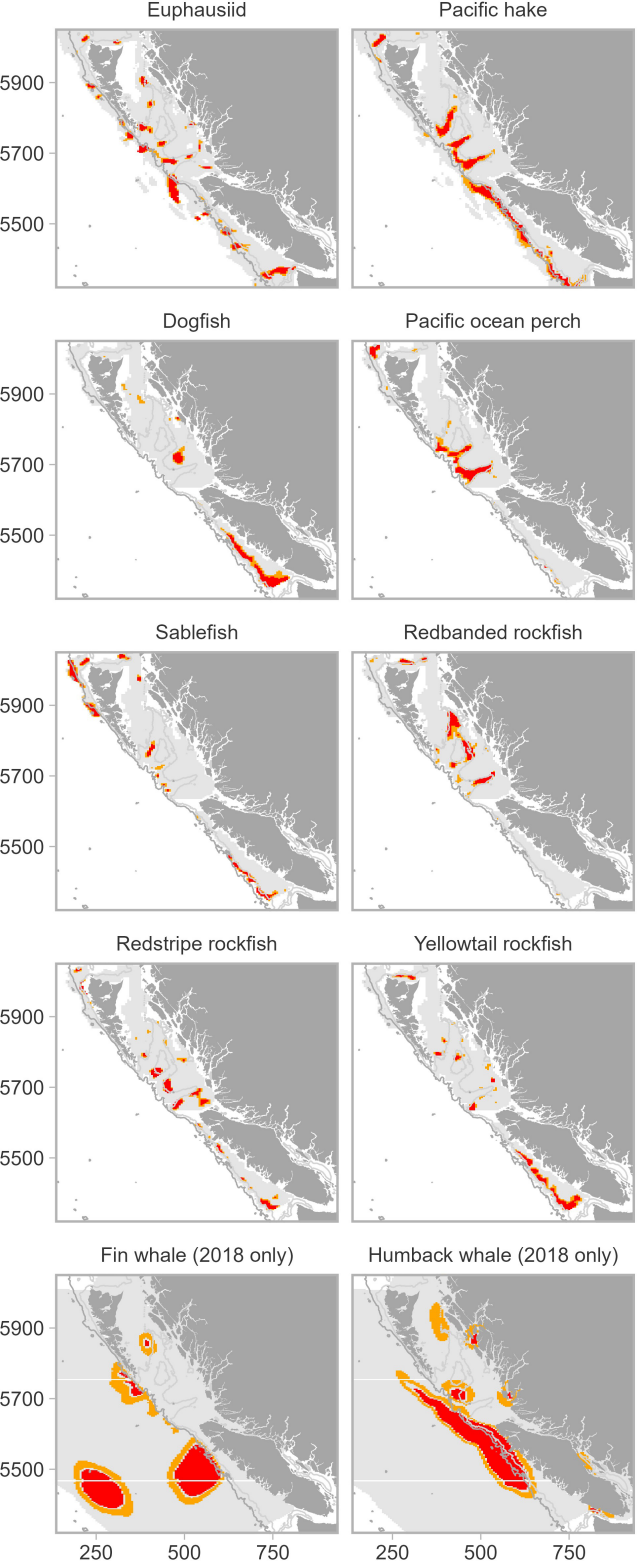
Distribution of biomass hotspots for each species which was the 90th percentile of *Z*‐scores calculated from predicted biomass using the Getis‐Ord statistic for each species. The spatial distribution of hotspots was quantified for 10 species for the Pacific coast of Canada and represent a mean across the years of sampling used to model the distribution of species—euphausiids and fish 2007–2018, whales 2018 only. Gray color on the shelf indicates surveyed area for each species.

**FIGURE 6:**
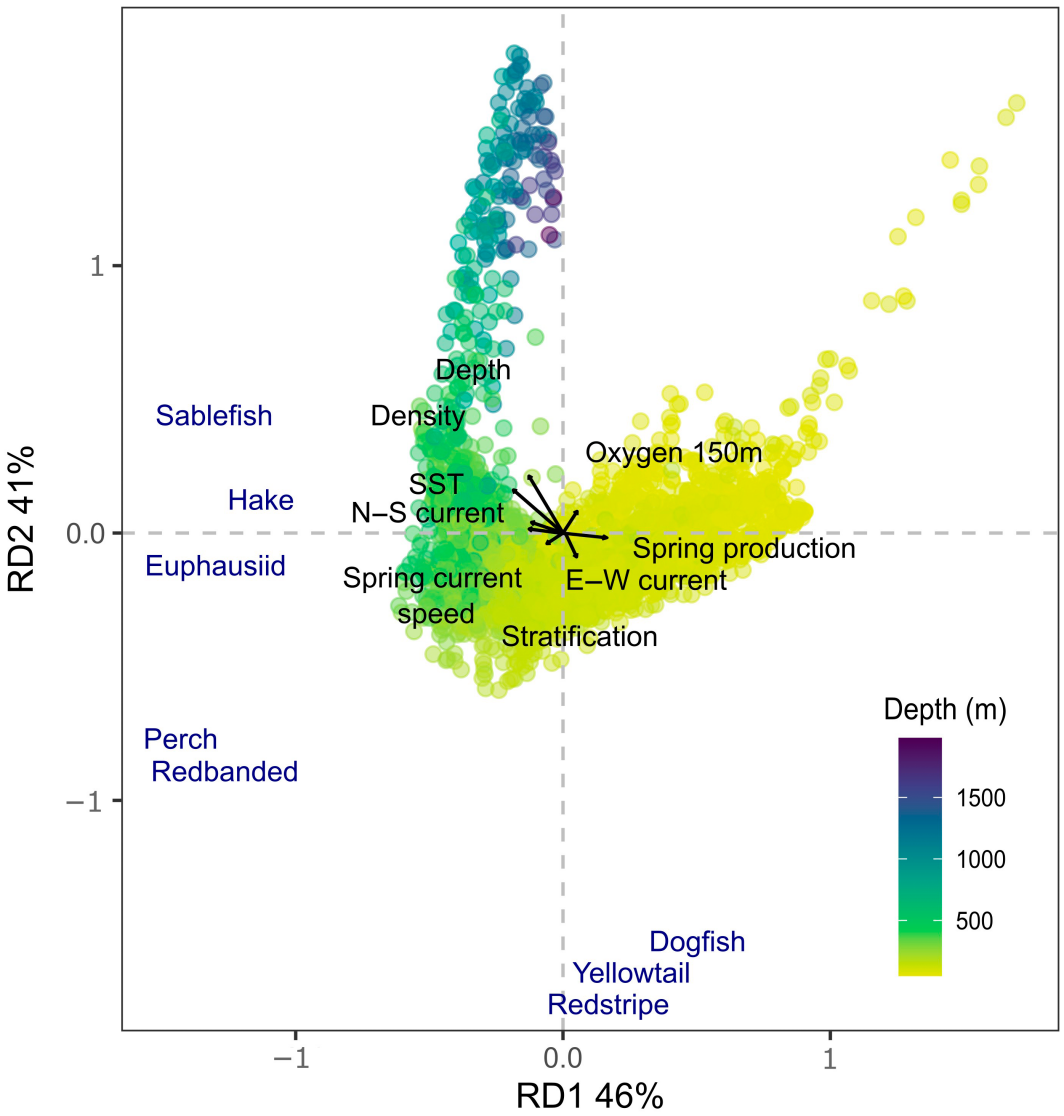
Redundancy analysis (RDA) triplot linking the spatial distribution (mean across 2007–2018) of *Z*‐scores (Getis‐Ord statistic) for fish and euphausiid species only with underlying environmental conditions. The underlying points used in the analysis are colored by depth (in meters) to show the depth‐gradient patterns in associations. Whales were excluded due to differences in temporal resolution.

Hotspots of sablefish co‐occurred with hake and euphausiids on the continental slope between the 200‐ and 1000‐m contours while rockfish, ocean perch, and dogfish often occurred along or shallower than the 200‐m contour (Figure 5). A RDA linking hotspots of fish and euphausiids (i.e., excluding whales) with environmental conditions indicated that both major axes of variation were associated with the coastal‐offshore depth gradient and explained a large amount of variation within the data (Figure 6). Bottom depth, water column density, and SST scored strongly and negatively in axis 1, while spring production scored positively (−0.5, −0.7, −0.5, and 0.5 respectively). Depth changes between 200 and 1000 m were the most important variable explaining axis 2, with all other variables scoring lower. Sablefish, hake, and euphausiids were part of a deeper water assemblage linked with lower water column density, the north–south current which flows along the continental slope and warmer SSTs away from coastal upwelling on the shelf. Sablefish were distributed deepest (RDA score along axis 2 of 0.45), then hake (0.27), with euphausiids found slightly shallower (−0.25) (Figure 6). Regions important for sablefish, hake, and euphausiids were also less stratified and higher in oxygen. In contrast, dogfish, yellowtail rockfish, and redstripe rockfish hotspots occurred in shallower coastal waters less than 200 m deep, which were cooler and more productive due to upwelling, represented by E–W current and production (Figures 5 and 6). These species were closely distributed along axis 1 (scores for axis 1: 0.2—redstripe, 0.4—yellowtail and 0.6—dogfish, respectively). The distribution of Pacific ocean perch and redbanded rockfish hotspots was also very similar, occurring only within the three sea valleys of Queen Charlotte Sound and north of Haida Gwaii in waters deeper than 200 m, but not reaching the continental slope margin at the 1000‐m contour (Figures 5 and 6). This was shown in the RDA with redbanded rockfish scoring −0.8 and Pacific ocean perch −0.7 along axis 2, which was heavily correlated with the depth gradient between 200 and 1000 m (Figure 6).

**FIGURE 7:**
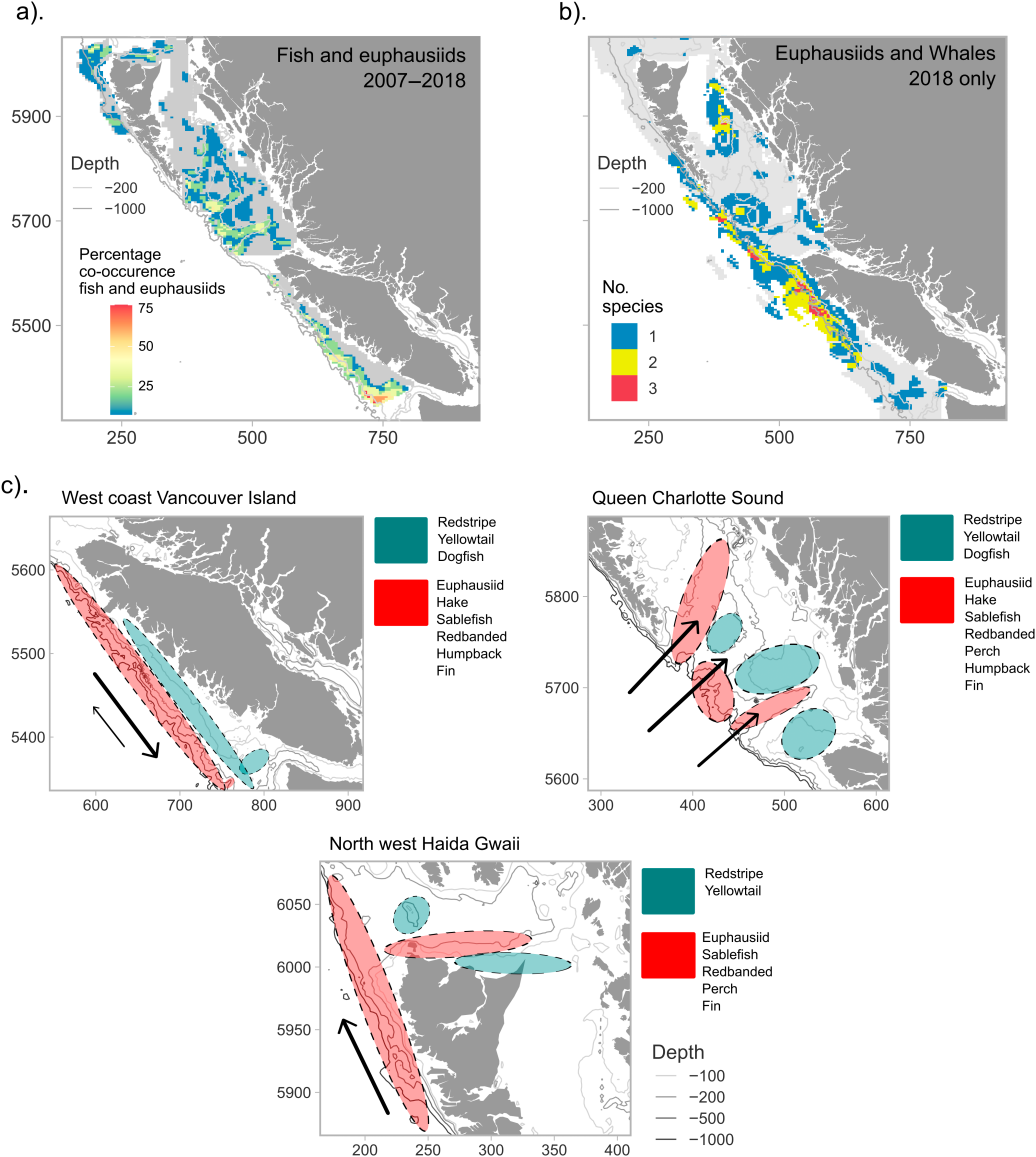
(a) The percentage co‐occurrence of biomass or density hotspots of euphausiids and seven species of fish modeled in the groundfish sampling area between 2007 and 2018, and (b) co‐occurrence of euphausiids with fin whales and humpback whales in 2018 only (area is truncated to the euphausiid sampling region as the smaller area but is analyzed at 5 × 5 km^2^ resolution to match the lower resolution of the whale data). Percentage co‐occurrence was calculated by counting how many species out of eight species of euphausiids and fish had *Z*‐values that fell in the 90th percentile for each grid square, therefore indicating hotspots. Gray color indicated smallest surveyed area which was analyzed (no hotspots were analyzed outside of this due to different species having different surveyed areas). (c) Three‐panel schematic focusing on the important ecological areas that are common between species. Different colors represent the depth‐gradient association of biomass hotspots between the two groups of species commonly found together. Black arrows indicate typical current direction and strength during summer, showing the shelf‐break current (large black arrow) and the Vancouver Island Coastal Current (small black arrow) on the left panel, the direction of upwelling onto the shelf (right panel) and Alaska Current on the bottom‐middle panel. For both, whale distribution only reflects patterns in 2018, while all other species represent predictions from 2007 to 2018.

Following a comparison of mean hotspot distribution for the eight species of fish and euphausiids that had multiyear data, we quantified areas with the highest mean co‐occurrence across species. Given that they were only surveyed in 2018, whales were not included in this comparative analysis. These areas were the west coast of Vancouver Island between the 200‐ and 1000‐m depth contours of the continental slope, and the La Perouse Bank region near the entrance of the Juan de Fuca canyon (Figure 7a). On average, >30% of the euphausiid and predator fish species exhibited *Z*‐scores in the 90th percentile in this region, with small areas of ~75% of species co‐occurring (Figure 7a). These included euphausiids, hake, redbanded rockfish, and sablefish along the deeper slope, and redstripe and yellowtail rockfish as well as dogfish along the shallower portion of the continental shelf (Figure 7c). Other areas with *Z*‐scores in the 90th percentile of biomass for 30%–50% of euphausiid and fish species analyzed were the deeper regions within two of the sea valleys of Queen Charlotte Sound. The sea valley closest to the northern tip of Vancouver Island exhibited the highest percent of co‐occurring hotspots (Figure 7a), with *Z*‐scores in the 90th percentile for euphausiids, hake, Pacific ocean perch, redbanded rockfish, and sablefish (Figures 5 and 7c). These sea valleys also supported both fin and humpback whales in 2018 (Figures 5 and 7b). In shallow areas (<200 m) between the sea valleys, hotspots of redstripe and yellowtail rockfish and dogfish were common. As along the west coast of Vancouver Island, species associations occurred along depth gradients as well as north of Haida Gwaii. The deepest areas in this region where the continental slope drops off steeply exhibited small hotspots for ~25% of species, particularly euphausiids, sablefish, redbanded rockfish, and Pacific ocean perch (Figures 5 and 7b). In this northern area, the shallower water redstripe and yellowtail rockfish hotspots were situated on the continental shelf northwest of Haida Gwaii in <200 m depth.

### Co‐occurrence between euphausiids and whales: 2018

In 2018 when humpback and fin whales were surveyed, hotspots were mostly concentrated over the continental slope and in the deeper oceanic waters off the shelf in similar areas to that of Pacific ocean perch, sablefish, redbanded rockfish, hake, and euphausiids (Figure 5). Both species were also concentrated along the northwest coast of Vancouver Island, while fin whales also had hotspots along the steep continental slope southwest of Haida Gwaii (Figure 5). We were able to quantitatively compare the distribution of hotspots of whales with euphausiids only, as euphausiids were the only other group with available data in 2018. Areas of co‐occurrence of the two whale species and euphausiids occurred along the continental slope off the north coast of Vancouver Island and along the continental slope at the start of the sea valleys in Queen Charlotte Sound (Figure 7b).

## DISCUSSION

Our study identified areas along the Pacific coast of Canada that, on average over the years analyzed, supported high densities of multiple key species for this ecosystem. This is critical information that could form the basis for ecosystem‐based management initiatives to maintain biodiversity, such as reducing vessel traffic in specific areas, dynamic fishery closures, or MPA designation (Ray, 2010). We found three key areas on the west coast of Canada with high co‐occurrence (30%–50%) of biomass hotspots for the species we analyzed—the continental shelf and slope area off the west coast of Vancouver Island, the sea valleys of Queen Charlotte Sound with their associated shallower areas, and the steep slope of the northwest coast of Haida Gwaii.

Studies spanning trophic levels across multiple species are challenging and complex, both at the data collection and the statistical modeling stage. Different types of survey data hold their own limitations and biases which are important to consider when interpreting the results, and the data used in this study relied on an array of different modeling techniques to mitigate some of these limitations. For example, hake are a large migratory stock (Beamish, 1981; Ressler et al., 2007), and therefore, our study encompasses only the portion of the stock which migrated into Canadian waters or is part of a resident population. We endeavored to use the best available data sources for each species modeled; however, given that the rockfish species, along with dogfish and sablefish, are often pelagic rather than obligatory bottom‐dwellers and were surveyed via a bottom trawl survey, biomass for these species might be underrepresented. In contrast to the survey data used for the other species, the whale data were from a single, large‐scale survey from summer 2018. These data are therefore a snapshot in time and some areas may be overrepresented, while some areas (e.g., the southwest coast of Vancouver Island) that are known to be quite important for these species have not been properly highlighted, due in large part to poor weather at the time of surveying. The acoustic data used for hake and euphausiids represent the best method currently available for surveying biomass density of these species. The spatiotemporal modeling approach we used for hake and euphausiids specifically accounts for many of the modeling challenges associated with field surveys with variable coverage across years (Anderson, Dunic, & English, 2024; Anderson, Dunic, Keppel, & Edwards, 2024; Webster et al., 2020). However, as with any regression model, any relationships found between biomass and the environment are correlative and not necessarily causative.

### Euphausiids and Pacific hake hotspot distribution

It is well known that complex bathymetry and edges associated with canyon systems and the continental shelf are extremely important factors in the development of euphausiid hotspots (Lavoie et al., 2000; Nickels et al., 2019; Santora et al., 2018; Schoenherr, 1991). These regions tend to occur adjacent to areas of strong alongshore shear and current velocity in areas of retention within features such as canyons that act to concentrate high coastal productivity as a result of upwelling (Lavoie et al., 2000; McManus & Woodson, 2012; Santora et al., 2018). These areas consequently become important resources for predators, particularly if persistent through time (Ainley et al., 2009; Benoit‐Bird et al., 2013; Diamond, 1981).

The extent of the northward migration of hake from California into Canada has been linked to temperature, with a greater proportion of the adult stock migrating farther north during warmer summers (Dorn, 1995; Malick et al., 2020; McFarlane et al., 1997; Smith et al., 1990). During the warm El Niño event of 1998, large numbers of adult hake migrated as far north as southern Alaska (54.5° N; Gauthier et al., 2016). However, our models indicated a bell‐shaped relationship between temperature at 150 m and abundance of hake off Canada, indicating that numbers of hake peaked at a particular temperature but decreased at higher temperatures. This could reflect the physical distribution of hake at the shelf break where upwelling occurs, with cooler, denser water being brought up the slope onto the shelf from the deep ocean further offshore. High biomass of hake is almost certainly located in this region to be adjacent to high biomass of euphausiids (Swartzman, 2001), and other small pelagic fish such as herring. Here, euphausiids displayed a negative relationship with temperature, preferring cooler water along the shelf break and on the shelf. As hake are a mainly migratory population, the abundance of hake that migrates into Canadian waters may also be dependent on conditions further south in the California Current, and the size structure of the stock, with larger individuals migrating further north (Malick et al., 2020). More recently, hake have been found to display a latitudinal relationship with temperature, with abundance being positively associated with warmer temperatures further south off California and off the west coast of Vancouver Island, but with cooler temperatures in the north off the coast of Haida Gwaii (Phillips et al., 2023), which could reflect differences in size distribution and preference of different ontogenetic stages (Malick et al., 2020).

The Juan de Fuca canyon is known to be an important region for both euphausiids and hake; however, we found lower hotspot overlap between these two species within the canyon and much more consistent overlap along the continental shelf off the west coast of Vancouver Island. This is consistent with another study in the region (Phillips et al., 2023), and with earlier studies emphasizing the importance of the shelf break for overlap of hake and euphausiid patches (Mackas et al., 1997; Ware & McFarlane, 1995). It is thought that when plankton patches are closer to the shelf break, they are easier for foraging hake to locate, reflecting optimal foraging theory (Swartzman, 2001; Townsend & Winfield, 1985). Two years with the lowest co‐occurrence (2007 and 2015) were both years of abnormal distributions of either hake or euphausiids; in 2007, hake abundance off the west coast of Vancouver Island was abnormally low and much higher in the north (King et al., 2012), and in 2015, euphausiid abundance showed the same pattern. These differences in distribution and the subsequent mismatch of predator and prey abundance may be related to large‐scale ocean processes; the La Niña event in late summer of 2007 led to lower than normal temperatures in the Gulf of Alaska and off California (McClatchie et al., 2008). An unprecedented MHW event occurred in the northeastern Pacific Ocean between 2014 and 2016 (Di Lorenzo & Mantua, 2016), resulting in changes to the structure and species composition of the zooplankton community from California to Alaska (Brodeur et al., 2019; Jones et al., 2018). There is much evidence that the distribution of hake, euphausiids and their overlap, is related to temperature and the size structure of the hake stock (Evans et al., 2023; Malick et al., 2020; Phillips et al., 2023; Swartzman, 2001), mediated by large‐scale ocean processes such as the El Niño–Southern Oscillation (ENSO). Mismatches in predator and preferred prey distribution, or a generally low abundance of preferred prey for many predator species, may lead to increased competition for resources and a greater expenditure of energy to locate alternatives. Smaller hake rely heavily on euphausiids while larger hake shift their diet toward fish, such as herring and other species (Tanasichuk et al., 1991). During 2015, hake off the west coast of Vancouver Island were small which may be why they did not migrate farther north (Malick et al., 2020). More work is required to ascertain if changing distribution patterns and differences in growth during extreme heatwave events are a direct result of environmental changes, or are due to changes in predation pressure, prey distribution, or competition.

### Spatial relationships and co‐occurrence of hotspots of euphausiids, fish, and whales

We found associations of species along the depth gradient of the continental shelf and slope that were determined by different oceanographic zones along the coast. This gradient links coastal waters with the wider ocean, and sea valleys and canyons situated along the boundary increase this connectivity. Three main areas of the coast were highlighted as ecologically important areas supporting hotspots of multiple euphausivorous species: the west coast of Vancouver Island concentrated along the Juan de Fuca canyon, the three sea valleys of Queen Charlotte Sound, including the shallow regions between valleys, and the northwest coast of Haida Gwaii. Species can co‐occur due to direct interactions such as predator–prey relationships or competition for the same resource. However, there are also other reasons for species co‐occurrence, including protection from predators while foraging or maximizing foraging efficiency while feeding on the same resource. For example, at small scales, salmon have been found to co‐occur with seabirds and facilitate prey capture by birds because they drive small forage species to the surface (Ballance et al., 1997; Harrison et al., 1991). Certain feeding behaviors of whales such as bubble‐net feeding or flick‐feeding may attract other predators by concentrating, stunning, or scattering prey (Ainley et al., 2009; Camphuysen & Webb, 1999; Diamond, 1981; Vaughn et al., 2008). This ecological phenomenon, among others, can lead to multispecies aggregations such as those observed at the mouth of the sea valleys in Queen Charlotte Sound.

On average through the years studied, five of the eight species analyzed co‐occurred in Queen Charlotte Sound, including euphausiids, hake, sablefish, redbanded rockfish, and Pacific Ocean perch. These species exhibited hotspots in the deeper waters of the sea valleys, which were also regions of hotspots of whales during the single‐year survey in 2018. These areas of co‐occurrence were concentrated along the Moresby Trough, a major conduit for upwelled oceanic water onto the shelf (Crawford et al., 1995; Ma, 1992; Whitney et al., 2005), and another deeper fjord system closer to the mainland known as Caamaño Sound, which exhibits high productivity due to tidal mixing (Clarke & Jamieson, 2006). Our results confirm that these were persistent areas of euphausiid hotspots across the years studied here. Co‐occurring hotspots of euphausiids, hake, dogfish, redstripe rockfish, sablefish, and yellowtail rockfish were present along the continental shelf break off the west coast of Vancouver Island and support previous work documenting the distribution of these species (Brodeur et al., 2009; Mackas et al., 1997; Swartzman, 2001). All species with hotspots in this region, except sablefish, hake and euphausiids, were distributed along the 200‐m bottom‐depth contour line. Sablefish growth exhibits a latitudinal gradient with larger individuals distributed in the more northerly part of their range such as along the Canadian coast, as well as an ontogenetic relationship with bottom depth with larger, older individuals found deeper than younger fish (Haltuch et al., 2019). Hake and euphausiid hotspots occurred in slightly more offshore waters between the 200‐ and 1000‐m bottom‐depth contour of the continental slope, with hake distributed adjacent to euphausiids over slightly deeper water. Previous work in this region found the shelf break to be an important area for hake predation on euphausiids (Mackas et al., 1997; Tanasichuk, 1999). Although we did not quantify the distribution of Pacific herring hotspots, herring are known to be present in highest densities between 50 and 150 m along the shelf break of the west coast of Vancouver Island (Godefroid et al., 2019). This co‐occurs with hotspots of all fish species analyzed in this study (except hake) and euphausiids, with herring a major part of the diet of several species including hake, dogfish and sablefish (Brodeur et al., 2009; Tanasichuk et al., 1991; Ware & McFarlane, 1986). Herring are known to display a negative distributional relationship with hake and euphausiids, thought to be due to predator avoidance of hake which are the most abundant fish on the west coast of Vancouver Island, or competition for the euphausiid resource (Mackas et al., 1997; McFarlane et al., 1997; Vestfals et al., 2023).

All hotspots in this study extended into the productive and bathymetrically complex Juan de Fuca eddy and canyon region off the southwest corner of Vancouver Island, which was a persistent area for nearly all the species analyzed. The Juan de Fuca canyon system is known to be one of the most productive areas of the whole northeast Pacific due to a combination of complex topography bringing nutrients onto the shallow waters of the continental shelf through the canyon and the eddy entraining these nutrients and the resulting production near the coast (Denman et al., 1981; Hickey & Banas, 2008; Ware & Thomson, 2005). The Swiftsure Bank area adjacent to the Juan de Fuca canyon is also an important area for humpback whales that was unfortunately not captured during the single‐year PRISMM survey (Wright et al., 2021), but the importance of this area year‐round for humpbacks has been documented in other studies (Dalla Rosa et al., 2012; McMillan et al., 2022).

## IMPLICATIONS AND CONCLUSIONS

We provide an integrated assessment of habitat use by key ecosystem and commercial species for one of the most productive regions for Canadian fisheries (Ware & McFarlane, 1986). Human influence along the whole west coast of Canada has greatly increased over the last few decades, with increased fishing pressures (Welch et al., 2021), and increased vessel traffic leading to increases in ship strike mortality for whales (Nichol et al., 2017). In addition, this region is exhibiting increased climatic variability as a result of anthropogenic climate change (Oliver et al., 2019; Sydeman et al., 2013). The integration of density and habitat‐use data for pelagic species provides a basis for identifying areas of missing information and for directing future research effort. Management strategies such as seasonal restrictions on vessel speeds in critical habitat areas supporting hotspots of multiple species, or seasonal closures for fishing during periods when multispecies aggregations occur to reduce bycatch of nontarget species could aid in mitigating the effects of climate and other anthropogenic stressors.

To directly support and develop dynamic management practices, more comprehensive and long‐term data are required. Our study does not evaluate differences in the vertical distributions of forage species; for example, which is greatly affected by changes in local current patterns associated with upwelling and is known to influence the distribution of predators. In addition, future work should focus on incorporating key species that are missing from this analysis, such as Pacific herring and other important species of small pelagic fish, which would provide the missing link between mid‐level predators and macrozooplankton. This has been done for the west coast of Vancouver Island (Godefroid et al., 2019), but is missing for the whole coast. Our study is the first fine‐scale assessment of the distribution of fish and whale hotspots relative to ocean climate and hotspots of euphausiids for the whole Canadian west coast and provides essential habitat‐use patterns for key species that can be used in support of ecosystem‐based management.

## AUTHOR CONTRIBUTIONS

Rhian Evans wrote the manuscript and conceived, designed, and carried out the analysis. Clifford L. K. Robinson and Stéphane Gauthier conceived of the idea and contributed to analysis design. Stéphane Gauthier and Chelsea Stanley collected the data. Stéphane Gauthier, Chelsea Stanley, Brianna M. Wright, Linda Nichol, and Philina A. English carried out part of the analysis and advised on analysis. All authors contributed to drafts.

## CONFLICT OF INTEREST STATEMENT

The authors declare no conflicts of interest.

## Supporting information


Appendix S1.

## Data Availability

Data for euphausiid and Pacific hake distribution models (Evans, 2025) are available in Zenodo at https://doi.org/10.5281/zenodo.16085479. Predicted distribution data for groundfish (English et al. 2022) are available in the Government of Canada's Open Government Portal under Record IDs 780a1c02‐1f9c‐4994‐bc70‐a0e9ef8e3968, 86af7918‐c2ab‐4f1a‐ba83‐94c9cebb0e6c, 557e42ae‐06fe‐426d‐8242‐c3107670b1de, and 5ee30758‐b1d6‐49fe‐8c4e‐5136f4b39ad1 at https://open.canada.ca/data/en/dataset/780a1c02‐1f9c‐4994‐bc70‐a0e9ef8e3968, https://open.canada.ca/data/en/dataset/86af7918‐c2ab‐4f1a‐ba83‐94c9cebb0e6c, https://open.canada.ca/data/en/dataset/557e42ae‐06fe‐426d‐8242‐c3107670b1de, and https://open.canada.ca/data/en/dataset/5ee30758‐b1d6‐49fe‐8c4e‐5136f4b39ad1, respectively. Predicted distribution data for whales (Wright et al., 2021) are available in the Government of Canada's Open Government Portal under Record ID: 39546277‐b33e‐4f80‐8a2d‐3ca1ce5b1401 at https://open.canada.ca/data/en/dataset/39546277‐b33e‐4f80‐8a2d‐3ca1ce5b1401.
