# Supplementary material for: Linking oceanic variability, euphausiid hotspot persistence, and marine predator distribution along Canada's west coast
Source: Ecol Appl. 2026 Jan 12;36(1):e70141. doi: 10.1002/eap.70141 (PMC12796558; doi:10.1002/eap.70141)
Supplement: Supplementary file 1 — Appendix S1. [file EAP-36-e70141-s001.pdf]

Appendix S1

Linking oceanic variability, euphausiid hotspot persistence, and marine predator distribution along Canada's west coast

Rhian Evans, Stéphane Gauthier, Clifford L.K. Robinson, Philina A. English, Chelsea Stanley, Brianna M. Wright, Linda Nichol

Ecological Applications

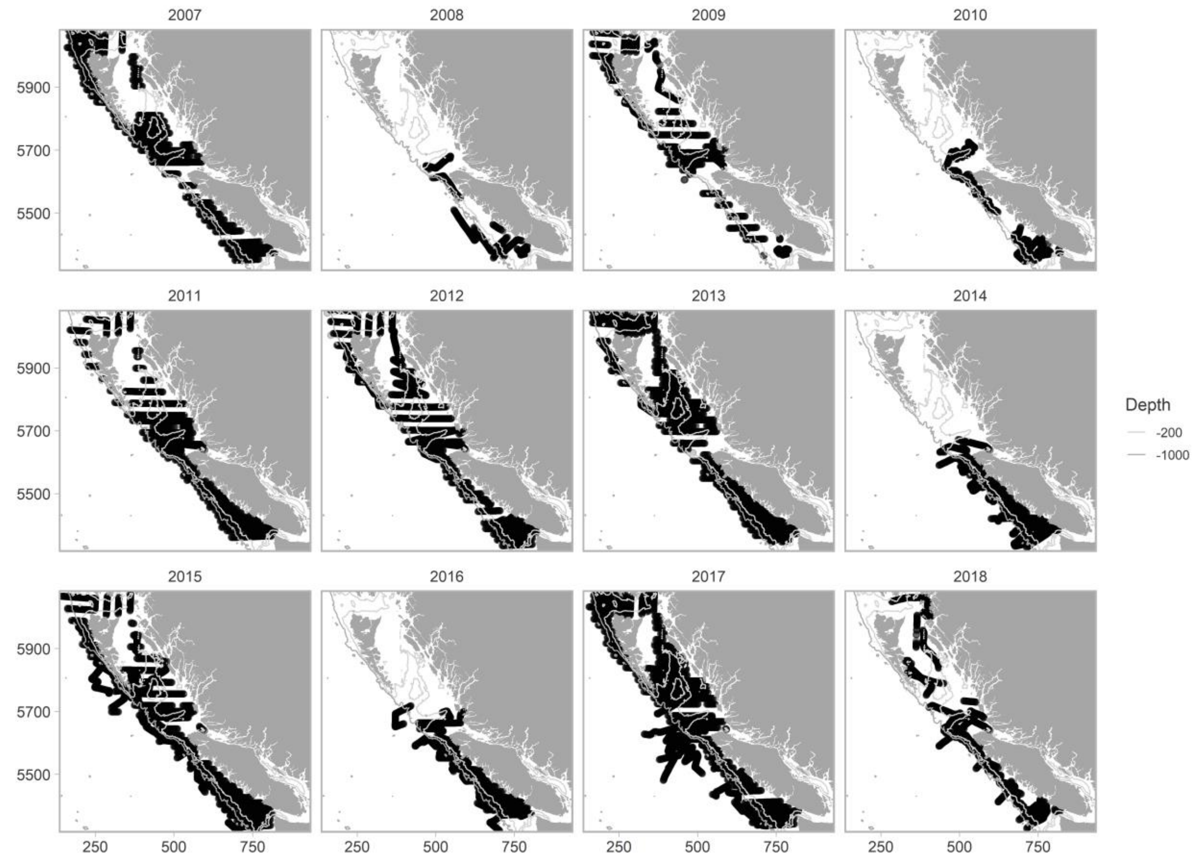

Figure S1. Distribution of euphausiid acoustic sampling transects by year.

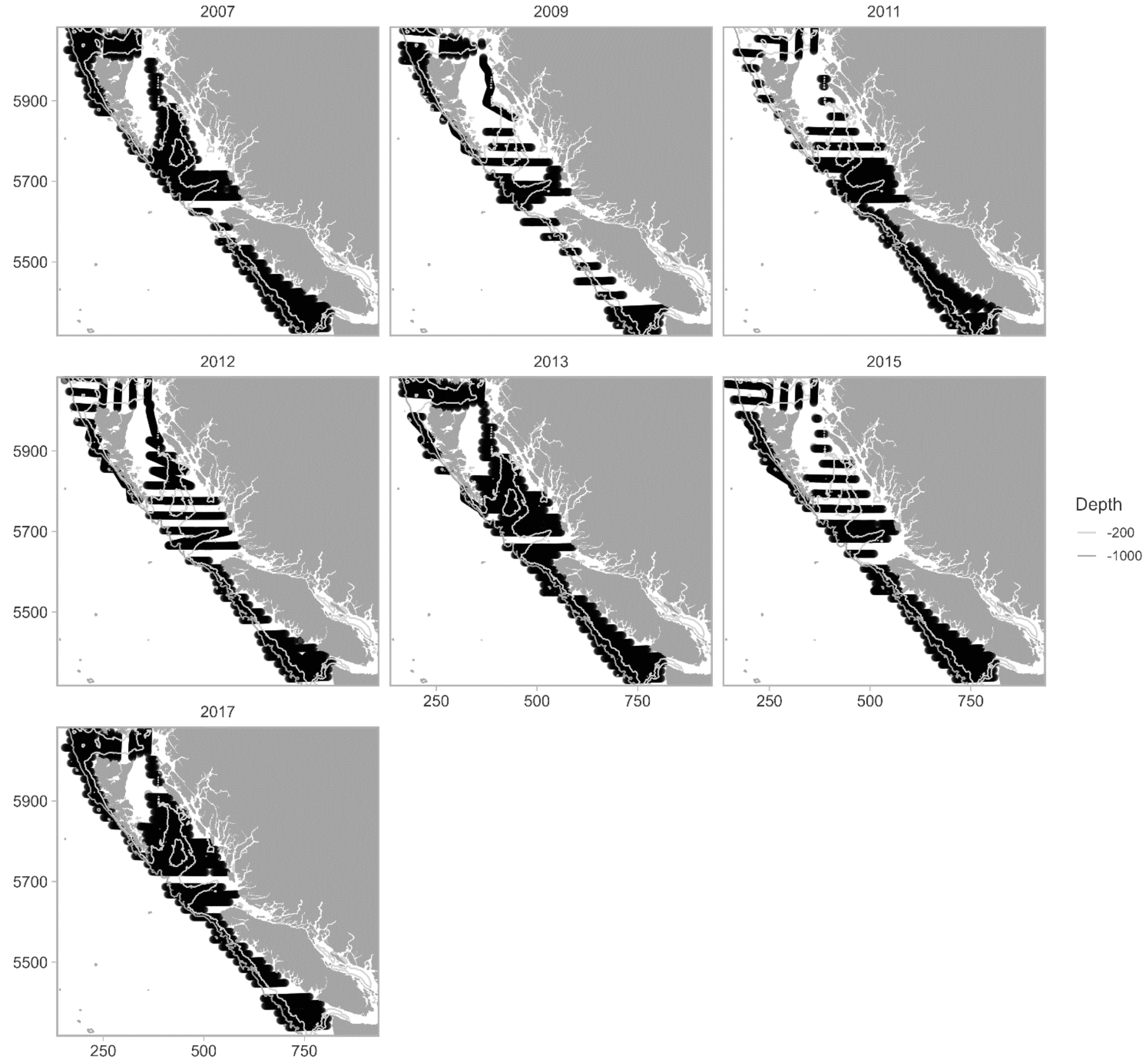

Figure S2. Distribution of Pacific Hake sampling transect by year.

Table S1. Model selection using uncorrelated sets of predictor variables for the August predictor and august euphausiid NASC (Aug/Aug model). Correlations existed within different forms of the same variables e.g. SST and temperature mean, however, correlations were also present between SST and production, temperature mean and density mean and V current and current speed. Some models did not converge and models with a log-likelihood gradient with respect to all fixed effects of >0.01 were discarded - NC indicates non-convergence of the model and so the AIC is not shown. Models in bold were considered the best models by AIC.

Model	Model	AIC
Aug / Aug model	Bathy, temperature mean, stratification, oxygen 150m, production phytoplankton, U current	NC
	Bathy, temperature mean, stratification, oxygen 150m, production phytoplankton, V current	421474.1
	Bathy, temperature mean, stratification, oxygen 150m, production phytoplankton, current speed	421477.6
	Bathy, temperature mean, stratification, oxygen 150m, production phytoplankton, U current, current speed	NC
	Bathy, temperature 150, stratification, oxygen 150m, production phytoplankton, U current	**421460.3**
	Bathy, temperature 150, stratification, oxygen 150m, production phytoplankton, V current	421489.3
	Bathy, temperature 150, stratification, oxygen 150m, production phytoplankton, current speed	NC
	Bathy, temperature 150, stratification, oxygen 150m, production phytoplankton, U current, current speed	**421461.6**
	Bathy, SST, stratification, oxygen 150m, U current	NC
	Bathy, SST, stratification, oxygen 150m, V current	421487.1
	Bathy, SST, stratification, oxygen 150m, current speed	NC
	**Bathy, SST, stratification, oxygen 150m, U current, current speed**	**421459.0**

Table S2. Model selection using May and August predictors for euphausiid NASC (see Table S1). For round one, only one May covariate was included in the model while the rest of the covariates tested were from August (one May/rest Aug), with the number of May to August predictors increasing until AIC began to increase. Correlations existed within different forms of the same variables e.g. SST and temperature mean, however, correlations were also present between SST and production, temperature mean and density mean and V current and current speed. Some models did not converge and models with a log-likelihood gradient with respect to all fixed effects of >0.01 were discarded - NC indicates non-convergence of the model and so the AIC is not shown. Models in bold were considered the best models in each round by AIC.

Model	Model	AIC
One May/ rest Aug	May SST	NC
	May stratification	421461.2
	May Oxygen 150m	NC
	May U current	**421459.8**
	May current speed	NC
Two May/ rest Aug	May SST and May stratification	421450.1
	May SST and May Oxygen 150m	NC
	May SST and May U current	421459.8
	May SST and May current speed	**421431.8**
	May stratification and May Oxygen 150m	421453.6
	May stratification and May U current	421471.2
	May stratification and May current speed	NC
	May Oxygen 150m and May U current	421459.5
	May Oxygen 150m and May current speed	NC
	May U current and May current speed	421457.6
Three May/ rest Aug	May SST and May stratification and May Oxygen 150	NC
	May SST and May stratification and May U current	NC
	May SST and May stratification and May current speed	NC
	May SST and May Oxygen 150 and May U current	421447.6
	**May SST and May Oxygen 150 and May current speed**	**421424.6**
	May SST and May U current and May current speed	NC
	May stratification and May Oxygen 150 and May U current	421463.0
	May stratification and May Oxygen 150 and May current speed	NC
	May stratification and May U surface and May current speed	NC
	May Oxygen 150 and May U surface and May current speed	NC

Table S3. Model selection using uncorrelated sets of predictor variables for the August predictor and august Pacific hake NASC (Aug/Aug model). Correlations existed within different forms of the same variables e.g. SST and temperature mean, however, correlations were also present between SST and production, temperature mean and density mean and V current and current speed. Some models did not converge and models with a log-likelihood gradient with respect to all fixed effects of >0.01 were discarded - NC indicates non-convergence of the model and so the AIC is not shown. Models in bold were considered the best models by AIC.

Model	Model	AIC
Aug / aug model	Bathy, temperature mean, stratification, oxygen 150m, production phytoplankton, U current	**55837.8**
	Bathy, temperature mean, stratification, oxygen 150m, production phytoplankton, V current	NC
	Bathy, temperature mean, stratification, oxygen 150m, production phytoplankton, current speed	55856.6
	Bathy, temperature mean, stratification, oxygen 150m, production phytoplankton, U current, current speed	NC
	Bathy, temperature 150, stratification, oxygen 150m, production phytoplankton, U current	55846.0
	Bathy, temperature 150, stratification, oxygen 150m, production phytoplankton, V current	55873.6
	Bathy, temperature 150, stratification, oxygen 150m, production phytoplankton, current speed	55858.4
	**Bathy, temperature 150, stratification, oxygen 150m, production phytoplankton, U current, current speed**	**55831.7**
	Bathy, SST, stratification, oxygen 150m, U current	55863.8
	Bathy, SST, stratification, oxygen 150m, V current	55897.9
	Bathy, SST, stratification, oxygen 150m, current speed	55880.9
	Bathy, SST, stratification, oxygen 150m, U current, current speed	55844.9

Table S4. Model selection using uncorrelated sets of predictor variables for the models with both May and August covariates for Pacific Hake. For round one, only one may covariate was included in a model with all other covariate from August, with the number of May to august predictors increasing until AIC began to increase. Correlations existed within different forms of the same variables e.g. SST and temperature mean, however, correlations were also present between SST and production, temperature mean and density mean and V current and current speed. Some models did not converge and models with a log-likelihood gradient with respect to all fixed effects of >0.01 were discarded - NC indicates non-convergence of the model and so the AIC is not shown. Models in bold were considered the best models in each round by AIC.

Model	Model	Gradient	AIC
One May/ rest Aug	May temperature 150m		55834.7
	May stratification		55825.1
	May Oxygen 150m		55833.5
	May production		**55799.2**
	May U current		55859.3
	May current speed		55846.4
Two May/ rest Aug	May temperature 150m and May stratification		55828.5
	May temperature 150m and May Oxygen 150m		55831.6
	May temperature 150m and May production		55799.2
	May temperature 150m and May U current		55867.3
	May temperature 150m and May current speed		55849.8
	May stratification and May Oxygen 150m		55828.2
	May stratification and May production		NC
	May stratification and May U current		55851.3
	May stratification and May current speed		NC
	May Oxygen 150m and May production		**55796.4**
	May Oxygen 150m and May U current		55864.6
	May Oxygen 150m and May current speed		55852.4
	May production and May U current		55819.7
	May production and May current speed		55804.8
	May U current and May current speed		55867.9
Three May/ rest Aug	May temperature 150m and May stratification and May Oxygen 150		55823.6
	May temperature 150m and May stratification and May production		55798.4
	May temperature 150m and May stratification and May U current		55856.7
	May temperature 150m and May stratification and May current speed		55844.2
	**May temperature 150m and May Oxygen 150 and May production**		**55795.7**
			55863.8

May temperature 150m and May Oxygen 150 and May U current	55848.9
May temperature 150m and May Oxygen 150 and May current speed	
May temperature 150m and May production and May U current	55829.7
May temperature 150m and May production and May current speed	NC
May temperature 150m and May U current and May current speed	55874.5
May stratification and May Oxygen 150 and May production	55796.6
May stratification and May Oxygen 150 and May U current	55855.4
May stratification and May Oxygen 150 and May current speed	55847.9
May stratification and May U surface and May current speed	55860.3
May Oxygen 150 and May production and May U current	55825.3
May Oxygen 150 and May production and May current speed	55810.4
May Oxygen 150 and May U current and May current speed	55874.8
May production and May U current and May current speed	55825.4

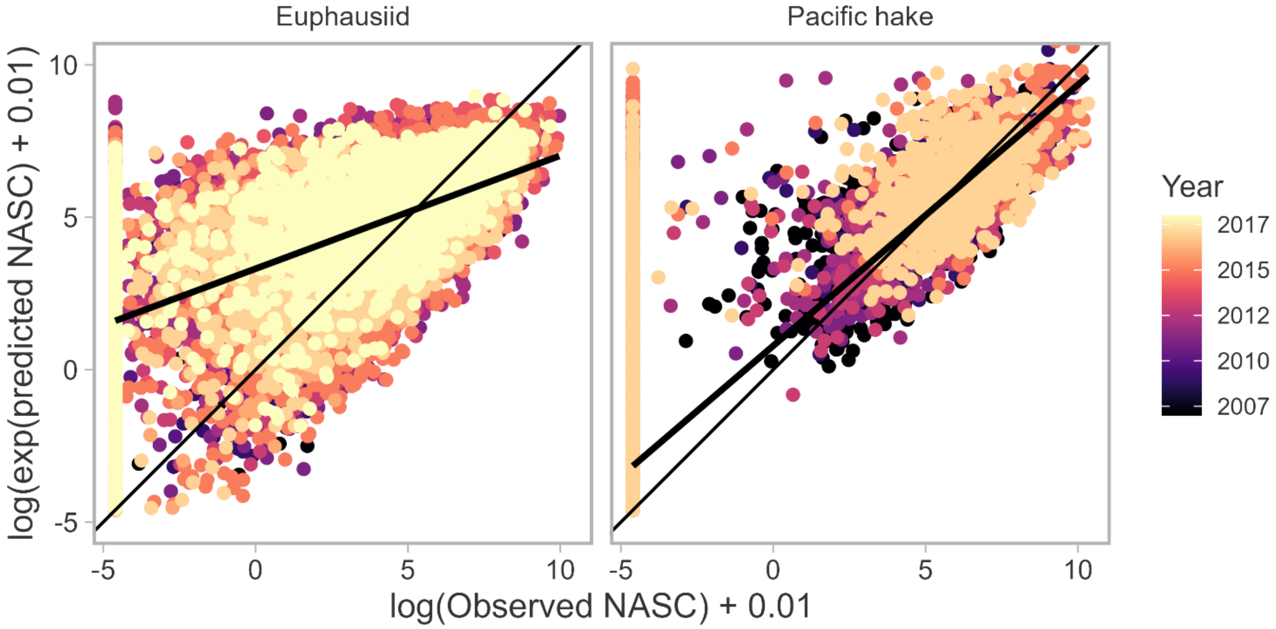

Figure S3. NASC predictions from the spatiotemporal models for a). euphausiids and b) Pacific Hake vs. observed sampled NASC from surveys. Observed biomass values were transformed via log(x+0.01) and predictions on the log-scale were transformed via log(exp(y) + 0.01). Both the correlation (thick black shorter line) and the 1:1 line (end to end diagonal line) are shown. The calculated model r^2 for the euphausiid model was 0.87, and for the hake model was 0.94.

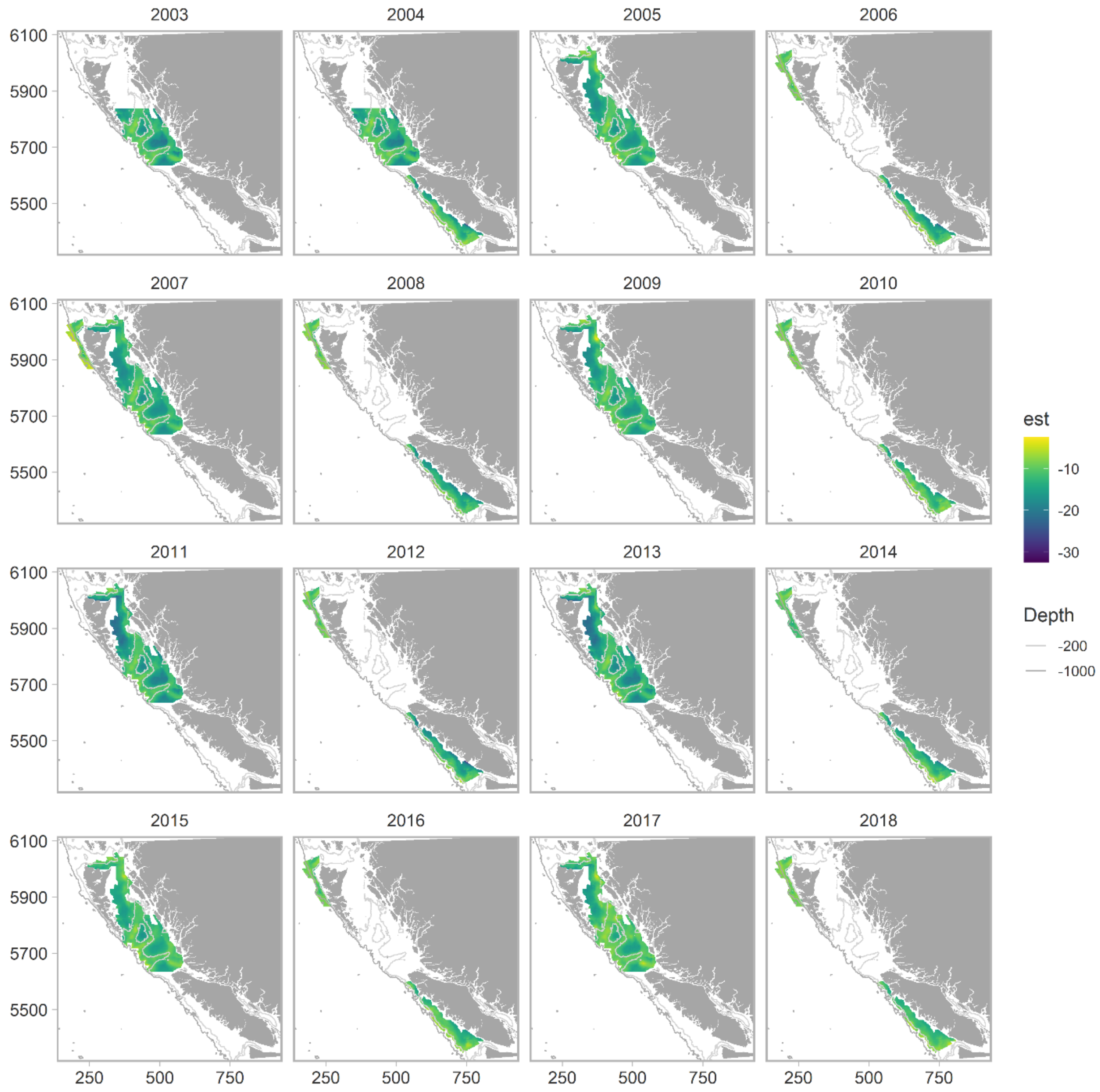

Figure S4. Example of predicted model domain for rockfishes, ocean perch, dogfish and sablefish using data from the DFO synoptic groundfish survey. These are predictions for Sablefish biomass from spatiotemporal modelling carried out by English et al. (2022). Fish density (est) is on the log-link-scale.

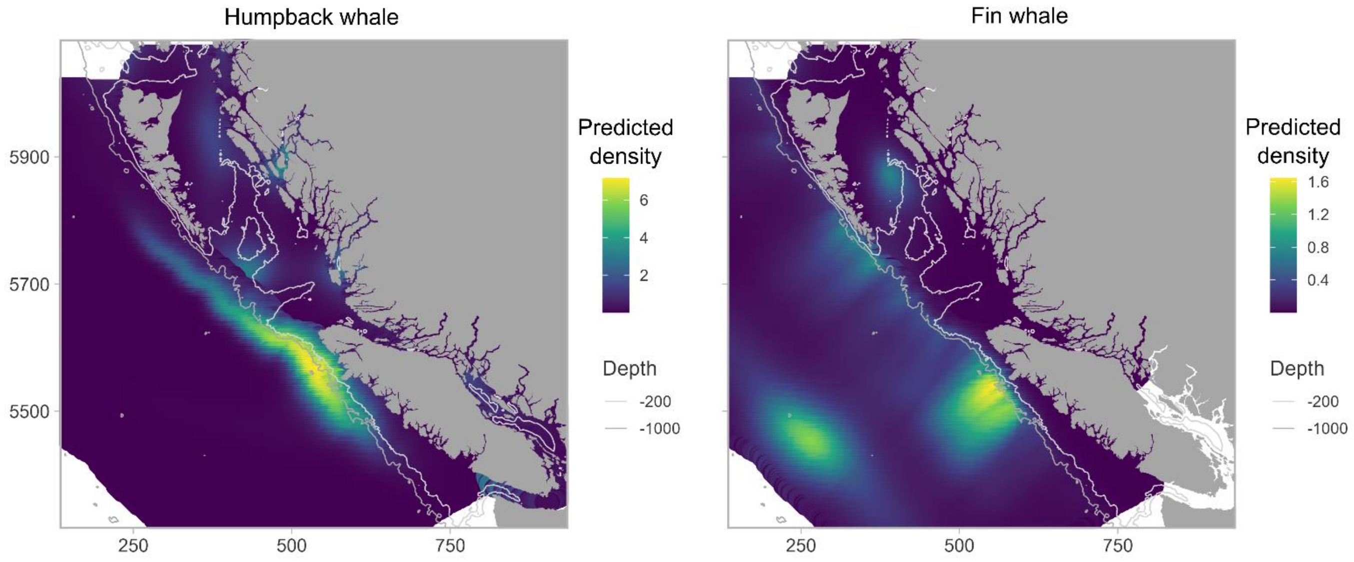

Figure S5. Predicted density (abundance per 25 km^2) of Humpback whales and Fin whales from the PRISMM survey carried out on the west coast of Canada. Please refer to Wright *et al.*, (2021) for a more detailed explanation of model development, structure and caveates.

References

English, P. A., E. J. Ward, C. N. Rooper, R. E. Forrest, L. A. Rogers, K. L. Hunter, A. M. Edwards, B. M. Connors, and S. C. Anderson. 2022. Contrasting climate velocity impacts in warm and cool locations show that effects of marine warming are worse in already warmer temperate waters. Fish and Fisheries 23:239–255.

Wright, B. M., L. M. Nichol, and T. Doniol-Valcroze. 2021. Spatial density models of cetaceans in the Canadian pacific estimated from 2018 ship-based surveys. Canadian Science Advisory Secretariat. Ottawa, Ontario, Canada.
